# Supplementary material for: Graph-based pangenomics maximizes genotyping density and reveals structural impacts on fungal resistance in melon
Source: Nat Commun. 2022 Dec 22;13:7897. doi: 10.1038/s41467-022-35621-7 (PMC9780226; doi:10.1038/s41467-022-35621-7)
Supplement: Supplementary file 1 — Supplementary Information [file 41467_2022_35621_MOESM1_ESM.pdf]

Graph-based pangenomics maximizes genotyping density and reveals structural impacts on fungal resistance in melon

Vaughn *et al.*

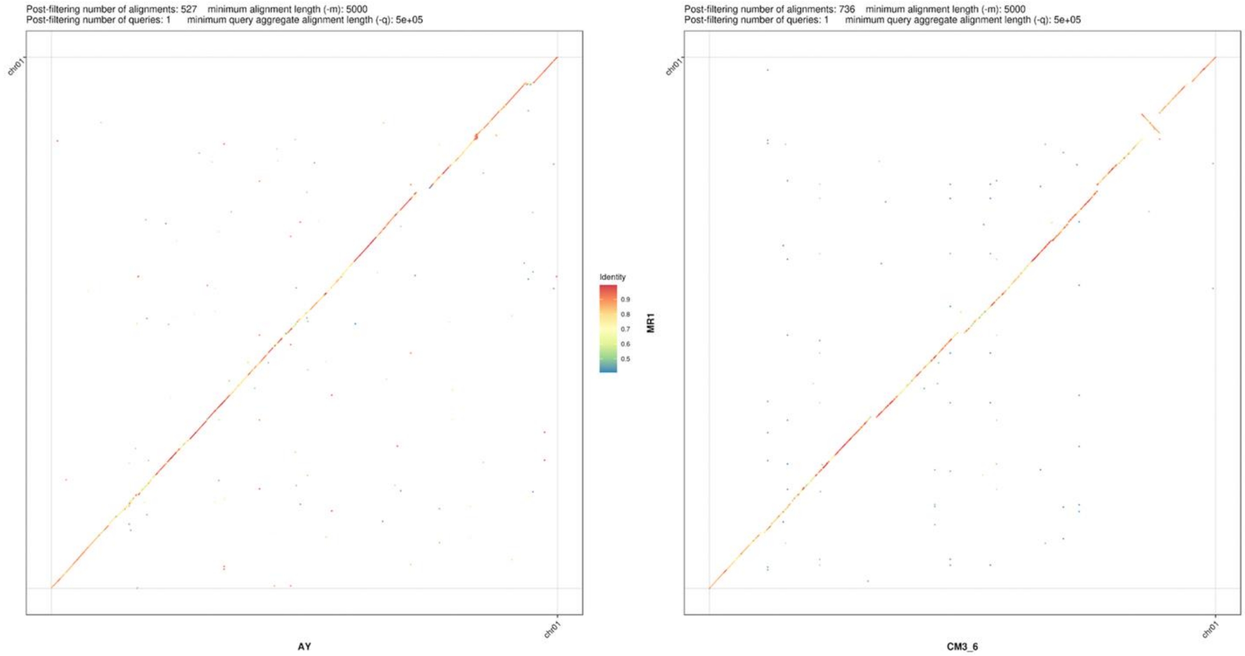

Supplementary Fig. 1. Dotplot of MR1 chromosome 1 relative to AY and to community reference DHL3.6. MR1 pseudomolecule is on y-axis in all panels; AY is the first column and DHL92 (also referred to its genome version as "CM3_6") is on the second column.

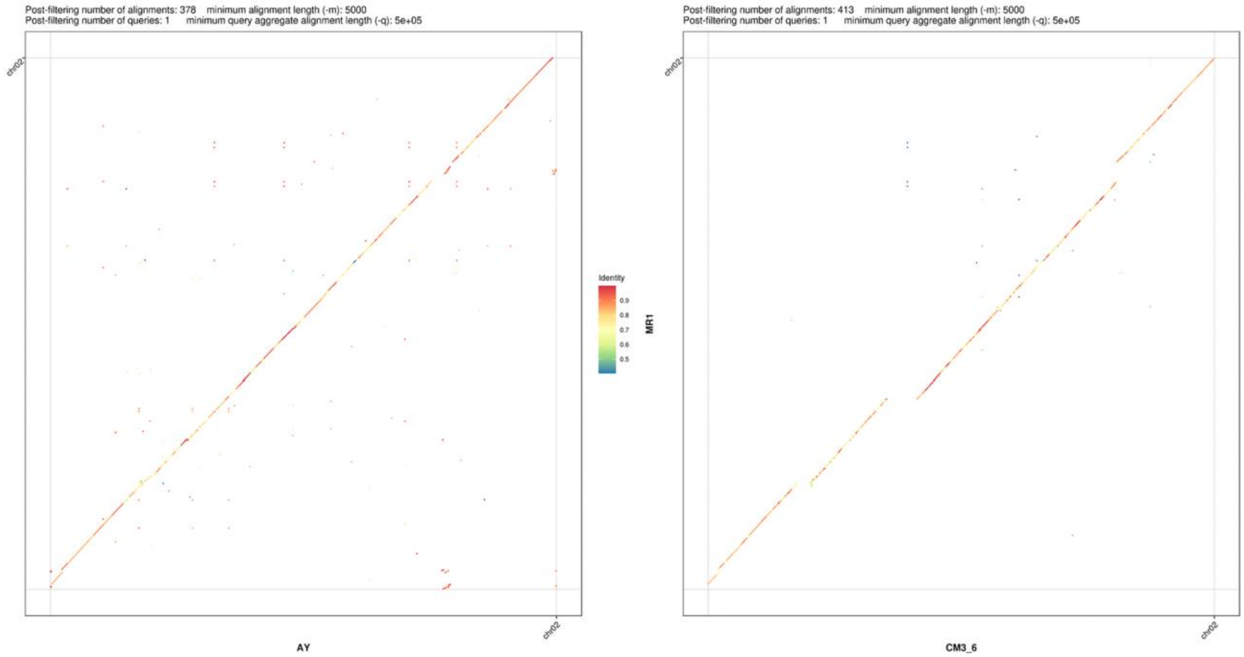

Supplementary Fig. 2. Dotplot of MR1 chromosome 2 relative to AY and to community reference DHL3.6. MR1 pseudomolecule is on y-axis in all panels; AY is the first column and DHL92 (also referred to its genome version as "CM3_6") is on the second column.

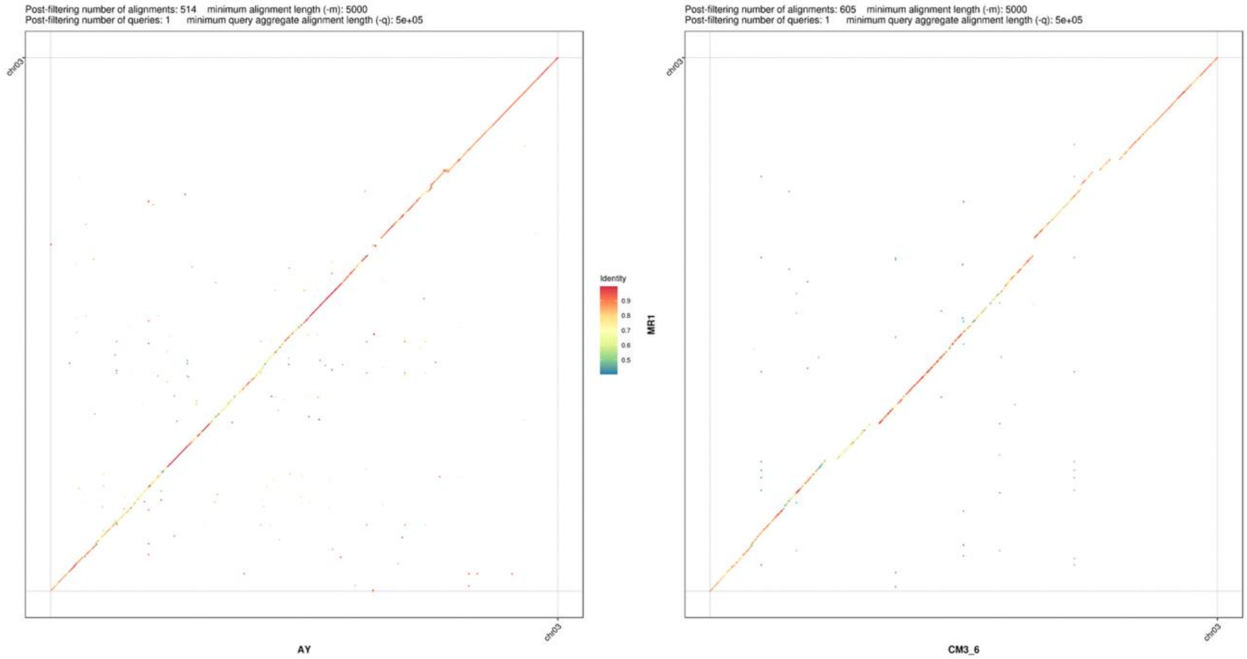

Supplementary Fig. 3. Dotplot of MR1 chromosome 3 relative to AY and to community reference DHL3.6. MR1 pseudomolecule is on y-axis in all panels; AY is the first column and DHL92 (also referred to its genome version as “CM3_6”) is on the second column.

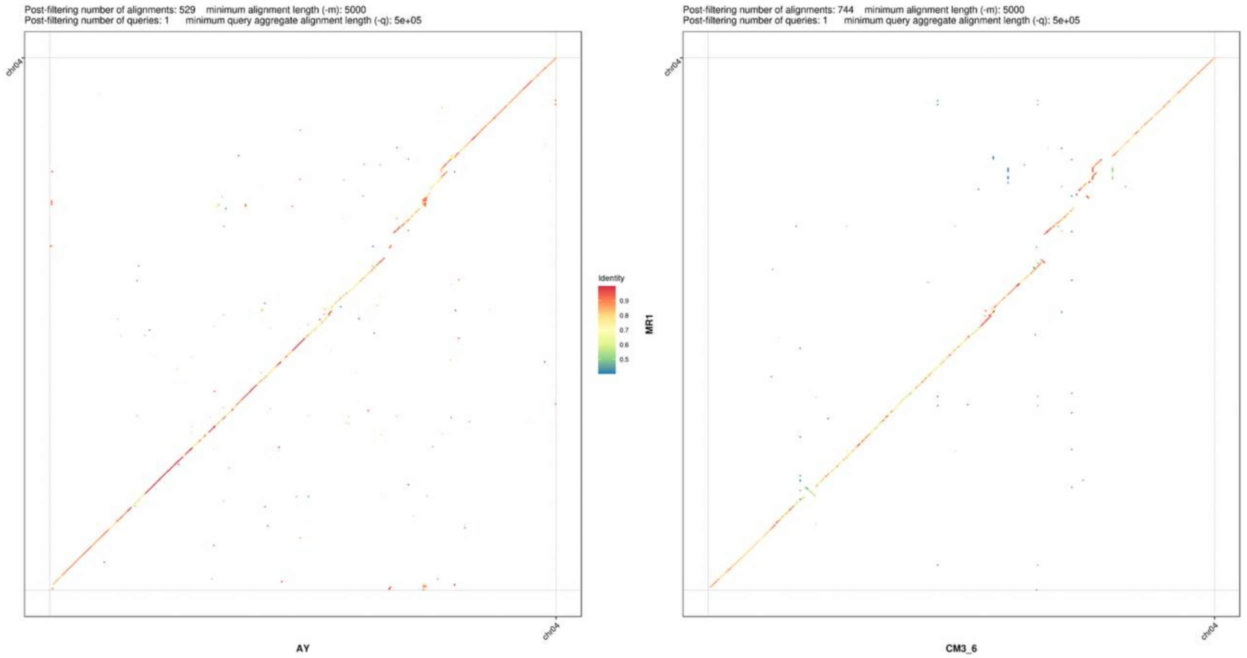

Supplementary Fig. 4. Dotplot of MR1 chromosome 4 relative to AY and to community reference DHL3.6. MR1 pseudomolecule is on y-axis in all panels; AY is the first column and DHL92 (also referred to its genome version as "CM3_6") is on the second column.

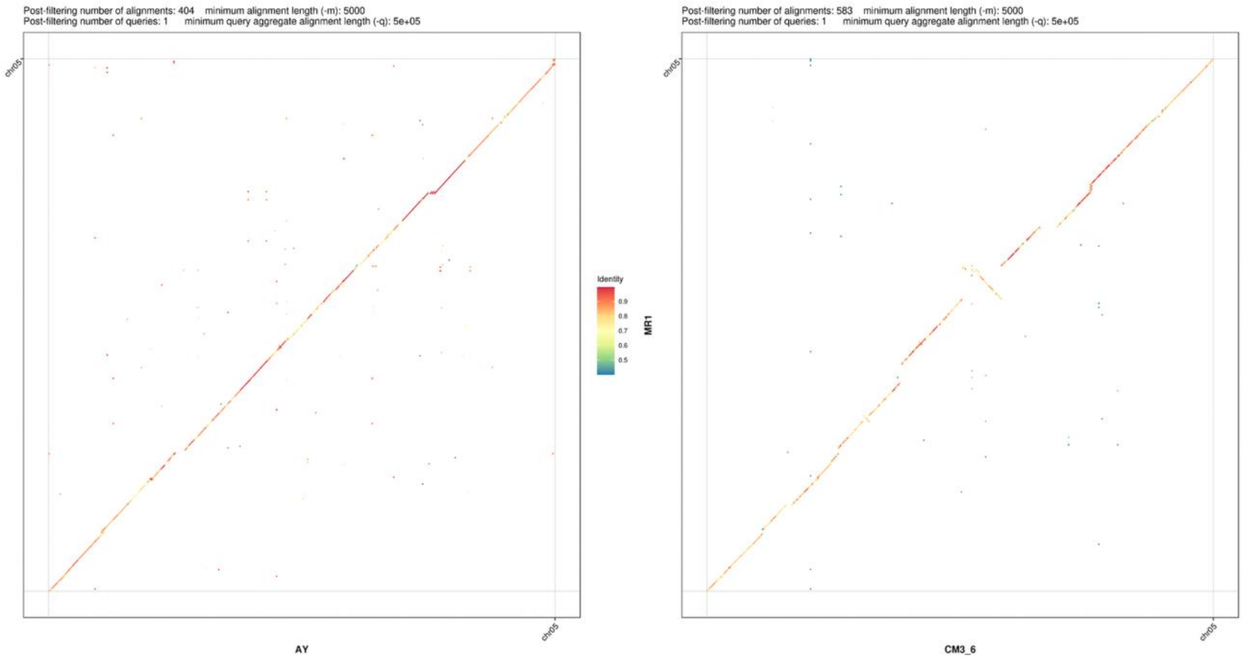

Supplementary Fig. 5. Dotplot of MR1 chromosome 5 relative to AY and to community reference DHL3.6. MR1 pseudomolecule is on y-axis in all panels; AY is the first column and DHL92 (also referred to its genome version as "CM3_6") is on the second column.

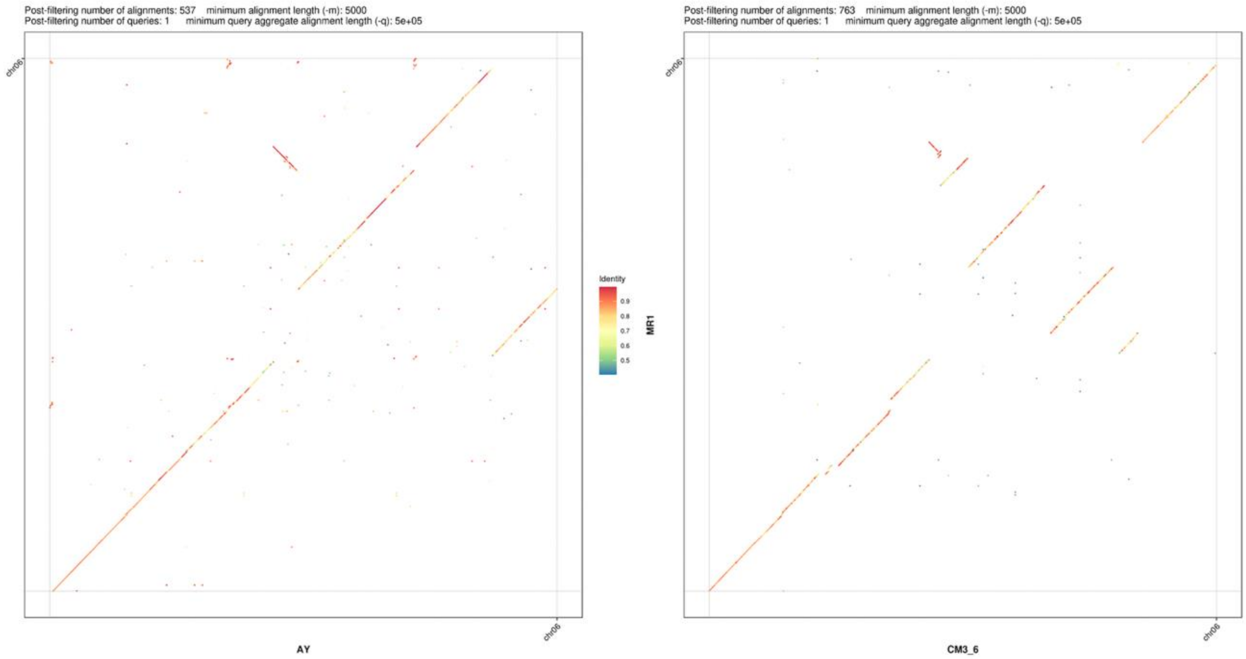

Supplementary Fig. 6. Dotplot of MR1 chromosome 6 relative to AY and to community reference DHL3.6. MR1 pseudomolecule is on y-axis in all panels; AY is the first column and DHL92 (also referred to its genome version as “CM3_6”) is on the second column.

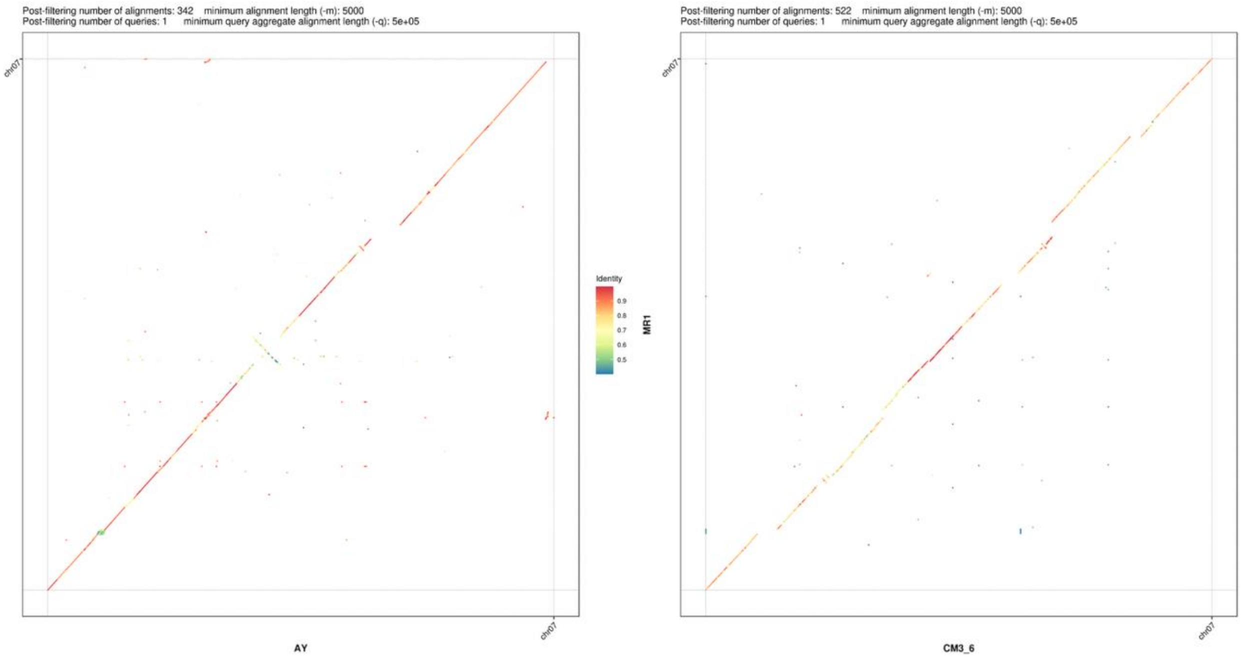

Supplementary Fig. 7. Dotplot of MR1 chromosome 7 relative to AY and to community reference DHL3.6. MR1 pseudomolecule is on y-axis in all panels; AY is the first column and DHL92 (also referred to its genome version as "CM3_6") is on the second column.

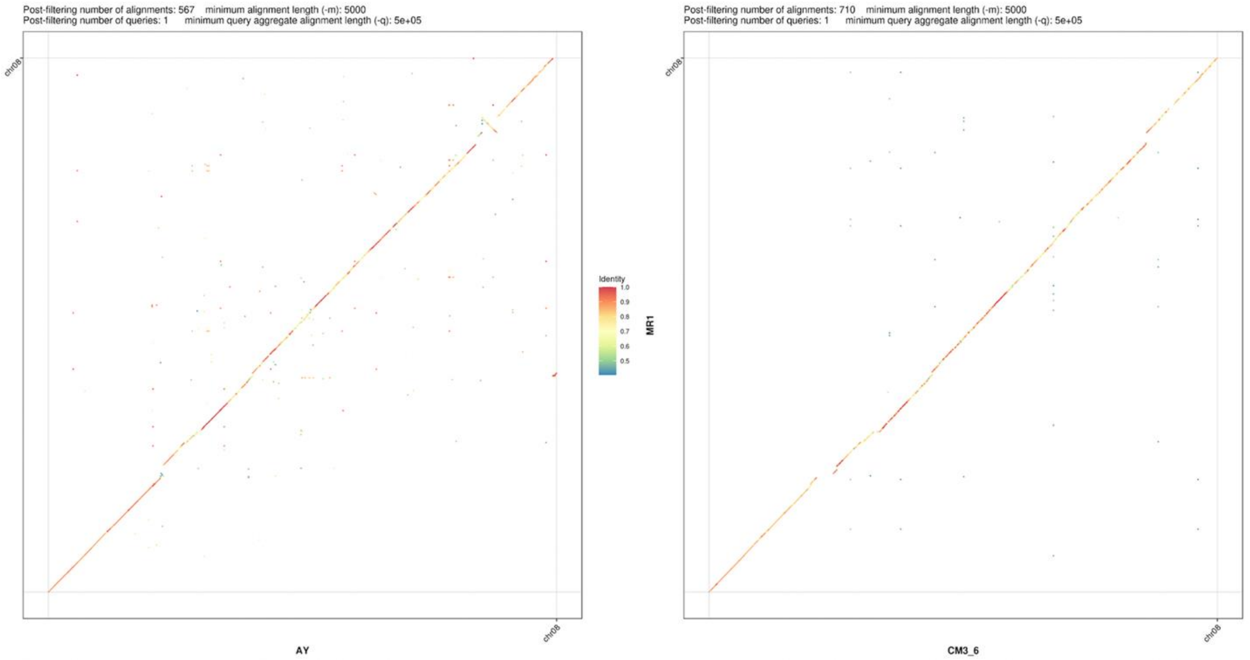

Supplementary Fig. 8. Dotplot of MR1 chromosome 8 relative to AY and to community reference DHL3.6. MR1 pseudomolecule is on y-axis in all panels; AY is the first column and DHL92 (also referred to its genome version as "CM3_6") is on the second column.

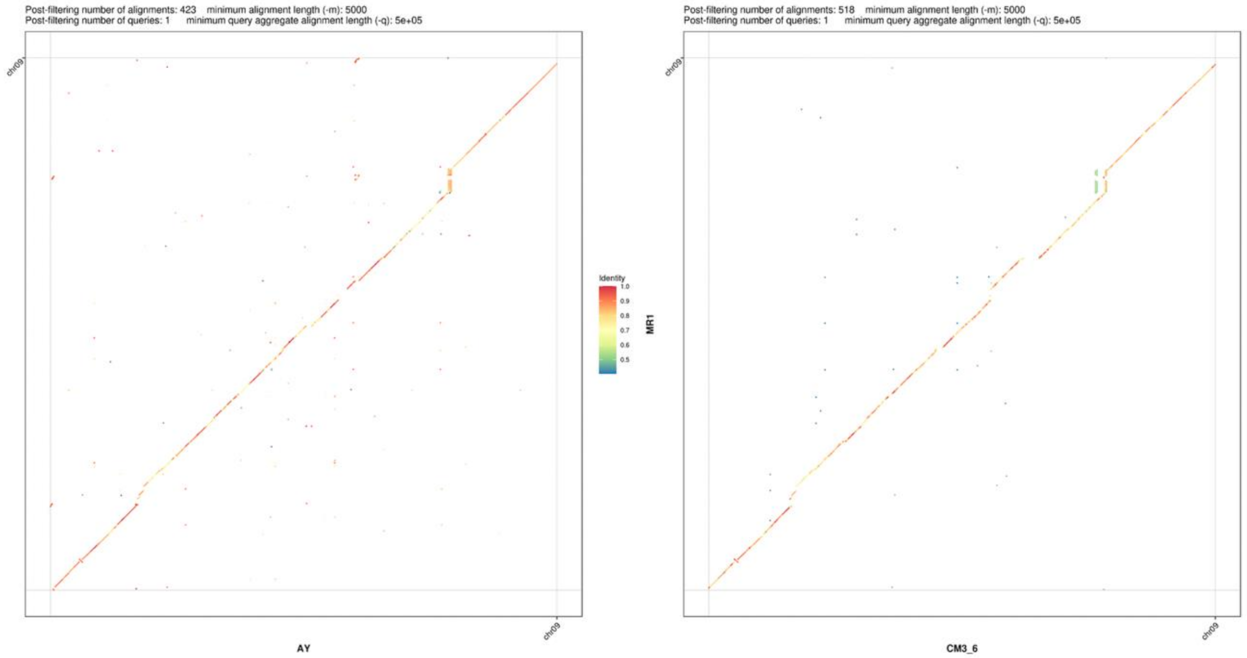

Supplementary Fig. 9. Dotplot of MR1 chromosome 9 relative to AY and to community reference DHL3.6. MR1 pseudomolecule is on y-axis in all panels; AY is the first column and DHL92 (also referred to its genome version as “CM3_6”) is on the second column.

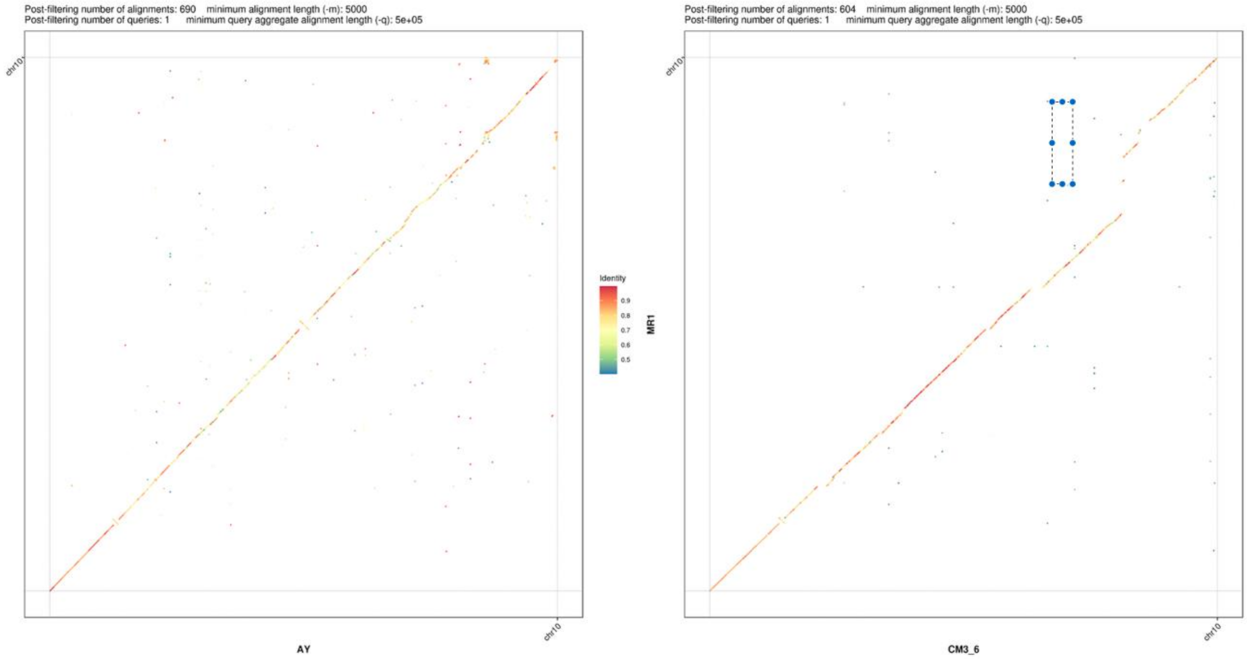

Supplementary Fig. 10. Dotplot of MR1 chromosome 10 relative to AY and to community reference DHL3.6. MR1 pseudomolecule is on y-axis in all panels; AY is the first column and DHL92 (also referred to its genome version as "CM3_6") is on the second column.

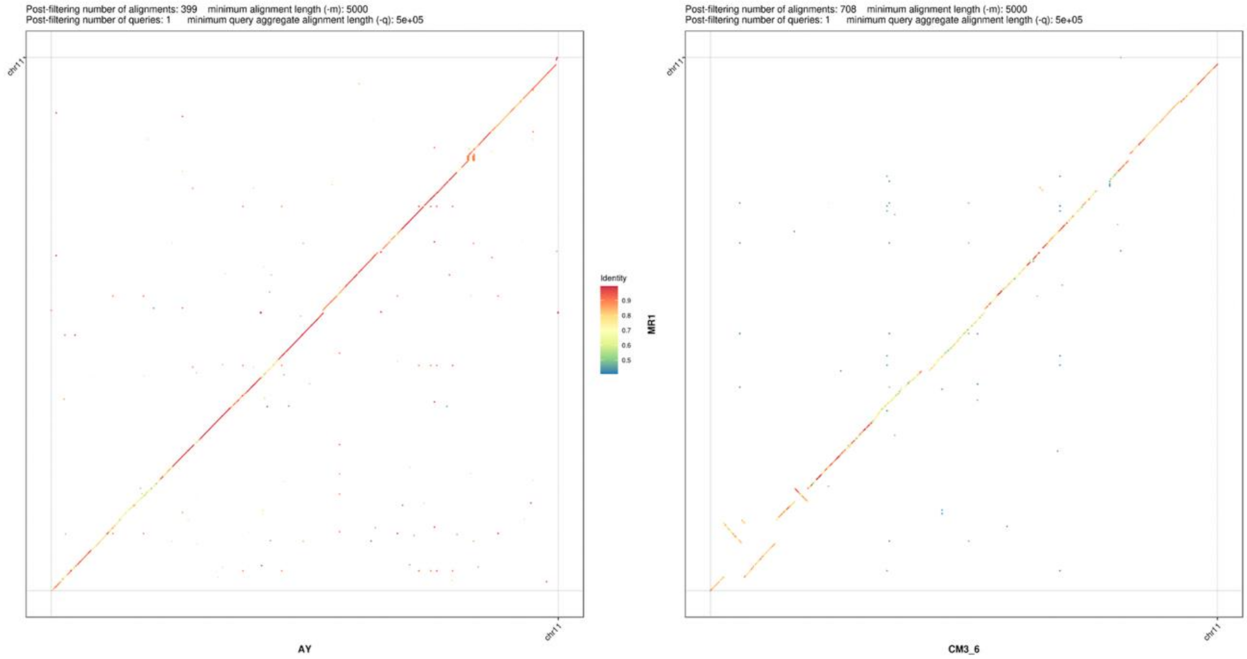

Supplementary Fig. 11. Dotplot of MR1 chromosome 12 relative to AY and to community reference DHL3.6. MR1 pseudomolecule is on y-axis in all panels; AY is the first column and DHL92 (also referred to its genome version as “CM3_6”) is on the second column.

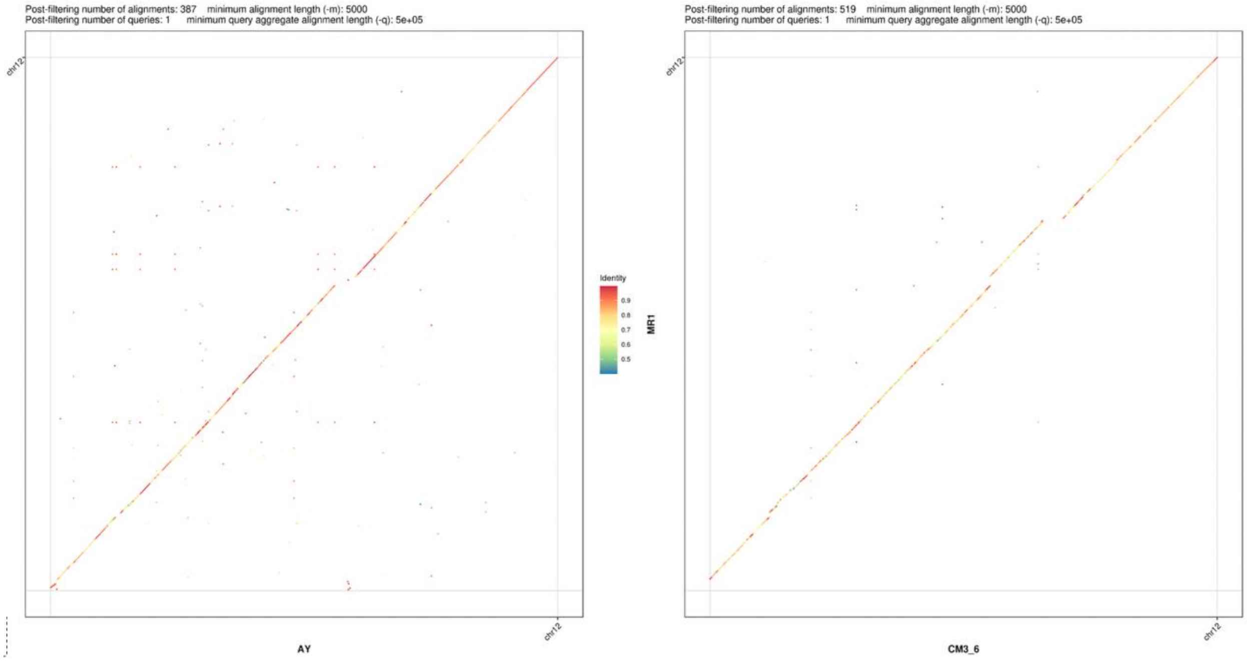

Supplementary Fig. 12. Dotplot of MR1 chromosome 12 relative to AY and to community reference DHL3.6. MR1 pseudomolecule is on y-axis in all panels; AY is the first column and DHL92 (also referred to its genome version as “CM3_6”) is on the second column.

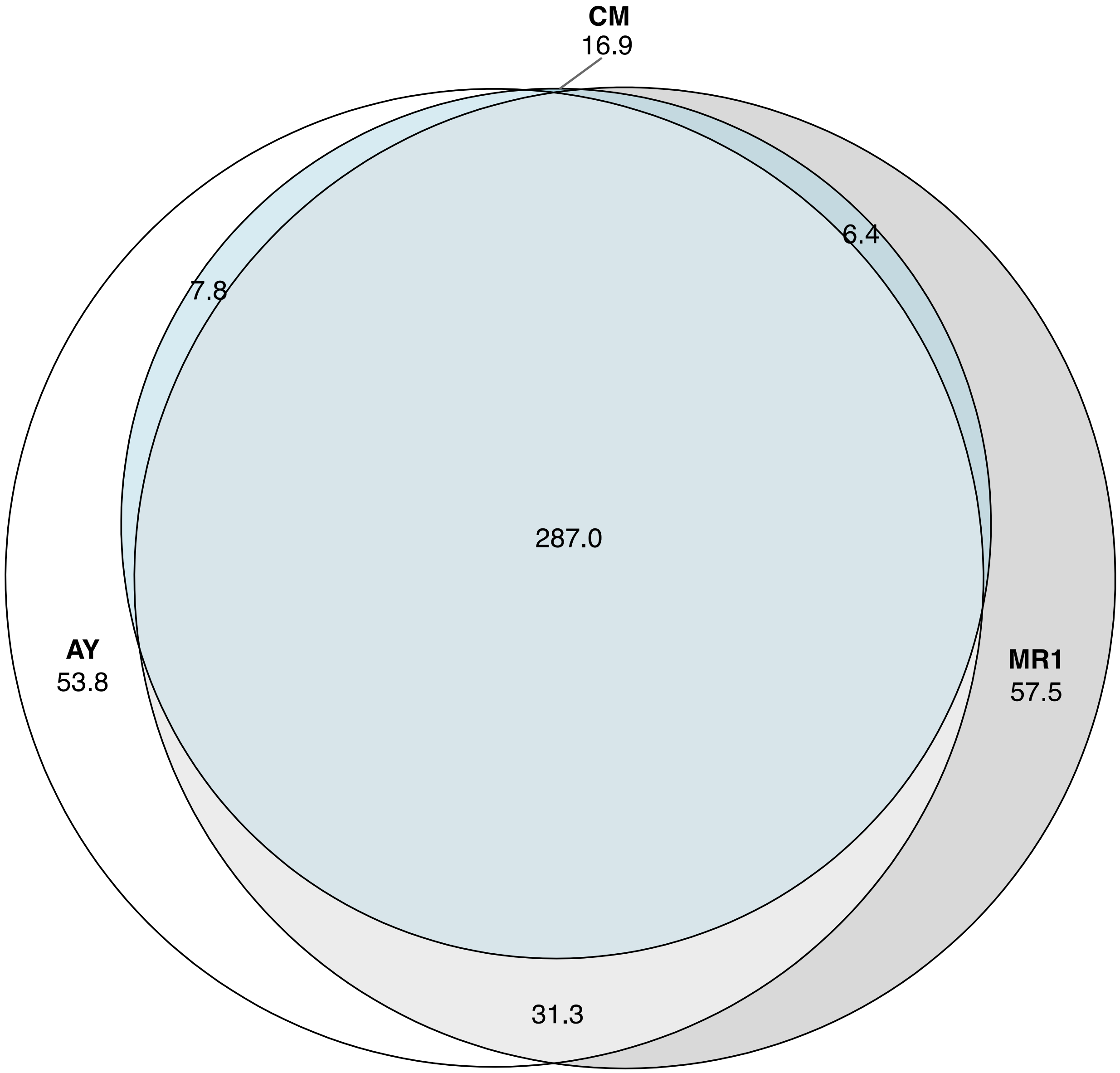

Supplementary Fig. 13. Venn diagram illustrating shared and unique nodes across the graph derived from MR1, AY, and DHL92 chromosome alignments. Overlaps are not perfectly proportional due to constraints on representation in circular form.

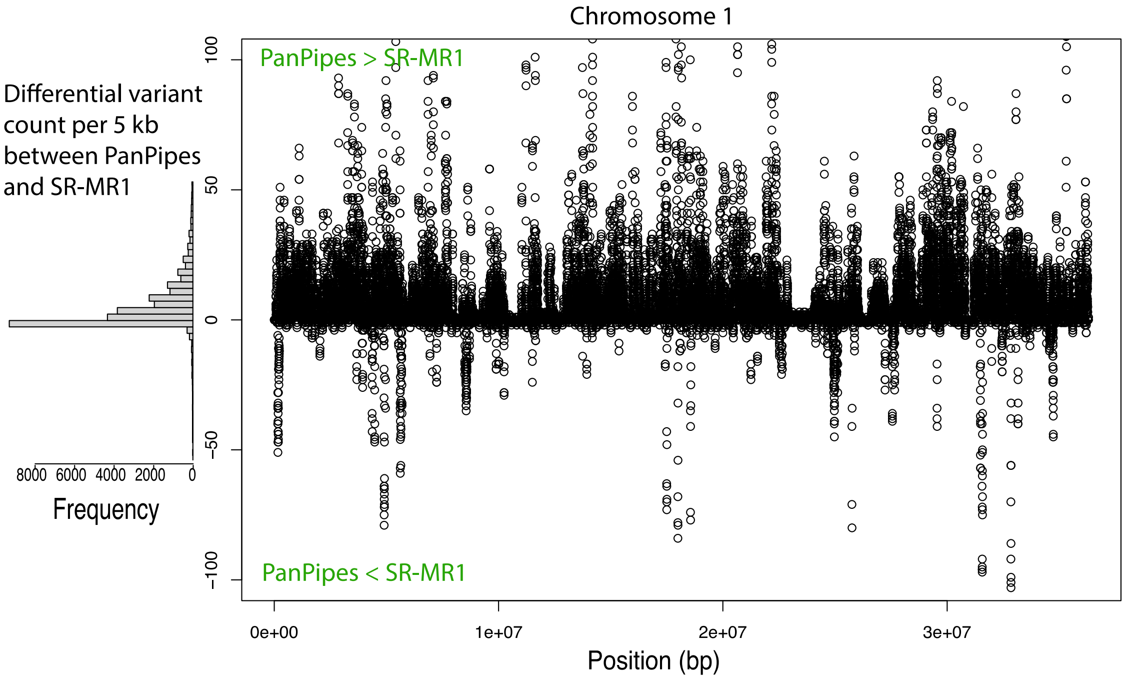

Supplementary Fig. 14. Variant density of PanPipes over SR-MR1 has a uniform spatial distribution. Using Chr 01 as an example and a 5000 kb window, the number of variants discovered (after filtering) is compared between the two methods. Positive values indicate a window for which PanPipes had more variants and the histogram of the y-axis values reflects that this surfeit is genome-wide and accounts for an additional 0.13 variants per 5 kb on average.

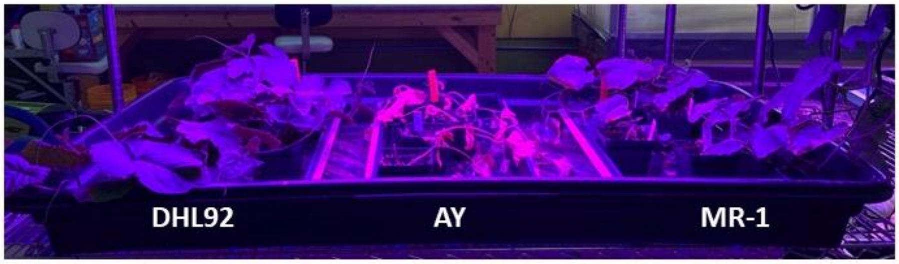

Plants inoculated with 1x10^6 conidia/ml suspension of *Fusarium oxysporum* f. sp. *melonis* race 1. Plants were grown for 21 days under a 16 hour photoperiod (LED lights) at 27°C after inoculation.

Supplementary Fig. 15. *Fusarium oxysporum* (race-1) wilt assay for MR1, AY, and DL92.

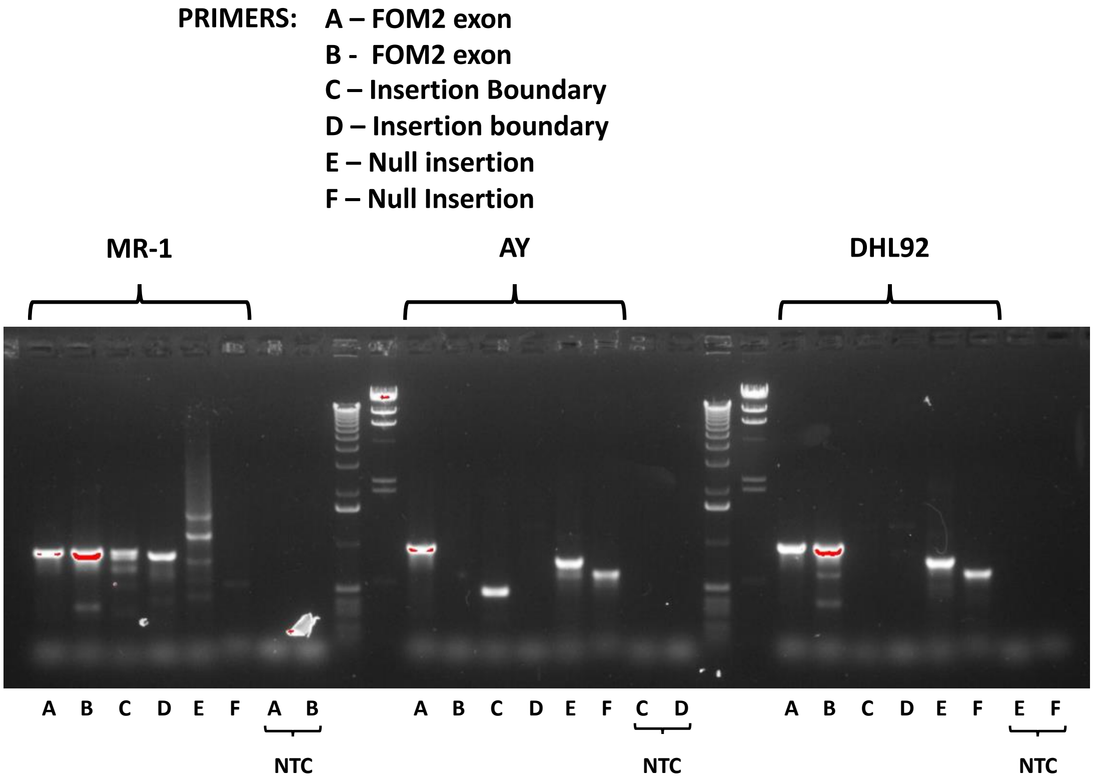

Supplementary Fig. 16. PCR confirmation on promoter/ORF haplotypes across MR1, AY, and DHL92. Primers A and B are replicates of two very similar regions of conserved portions of the NBS-LRR protein. B was a failed reaction in AY. Primers C and D are replicate primers that amplify the MR1 promoter insertion. Primer E and F are replicate primers for the sequence without the insertion. Primers C, D, E, and F required one side of each pair to fall in repetitive regions and so mis-amplifications are observed: C in AY and E in MR1 produce products but both are improperly sized and/or smeared. NTC, no-template controls

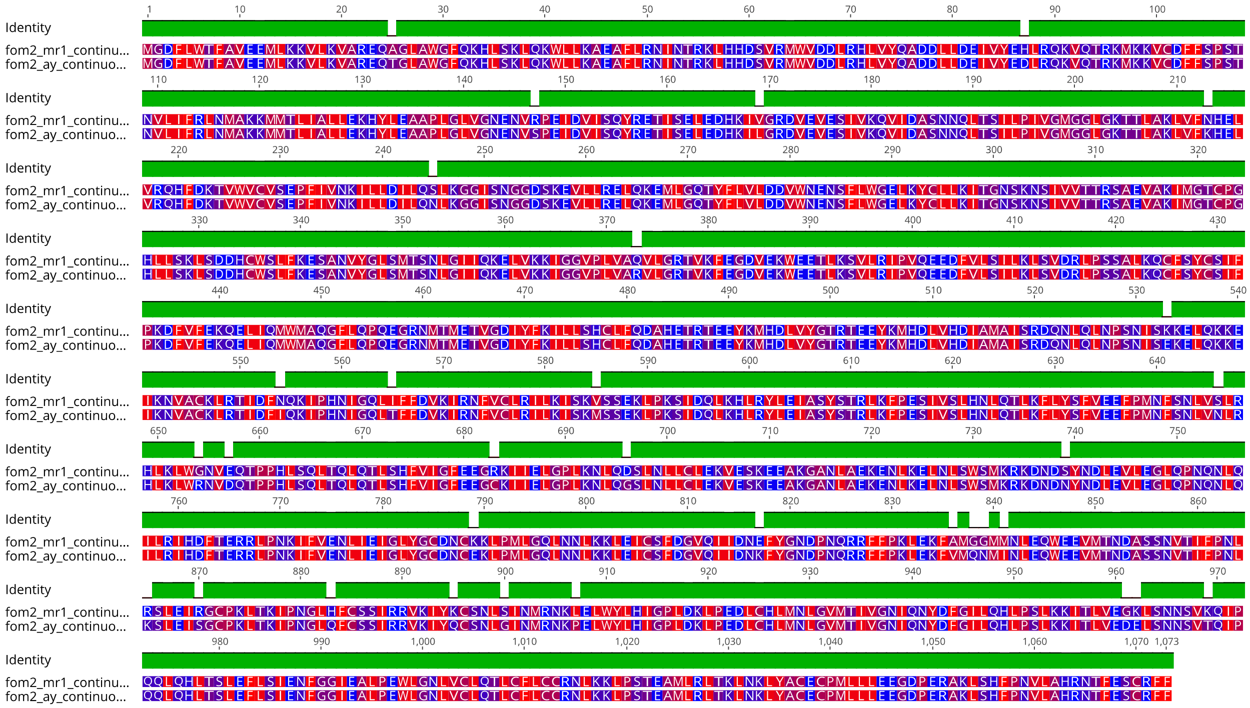

Supplementary Fig. 17. Protein alignment of *fom2* NBS-LRR. Color coding is based on hydrophobicity index with red being hydrophobic and blue being hydrophilic.

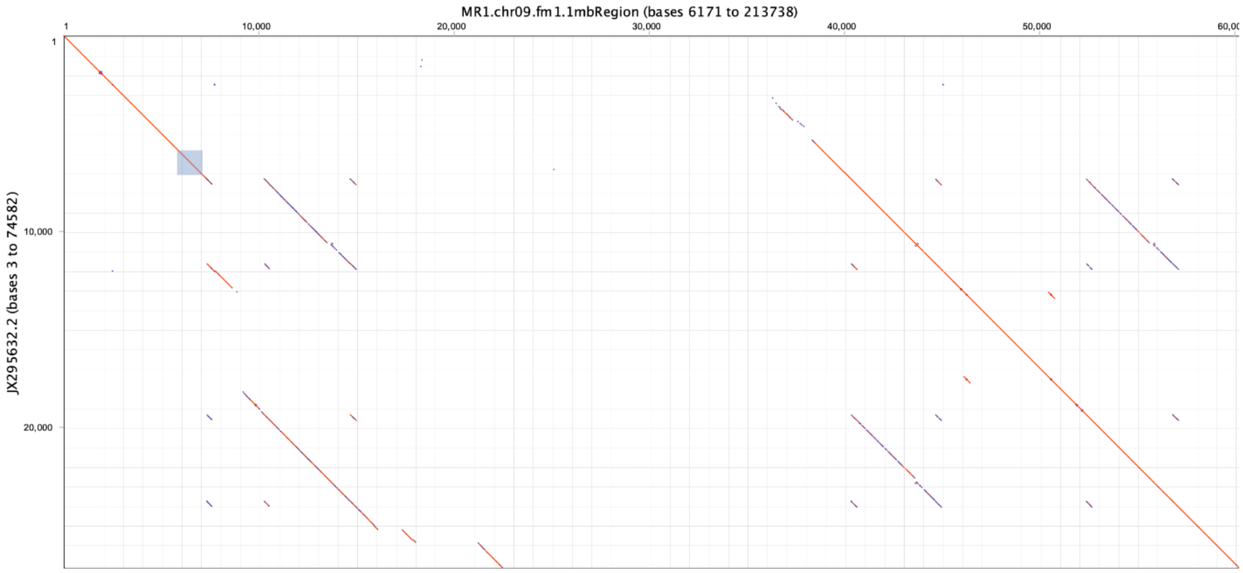

Supplementary Fig. 18. MR1 assembly (x-axis) versus previously deposited MR1 BAC JX295932.2 (y-axis). Blue highlighting represents *fom1* region.

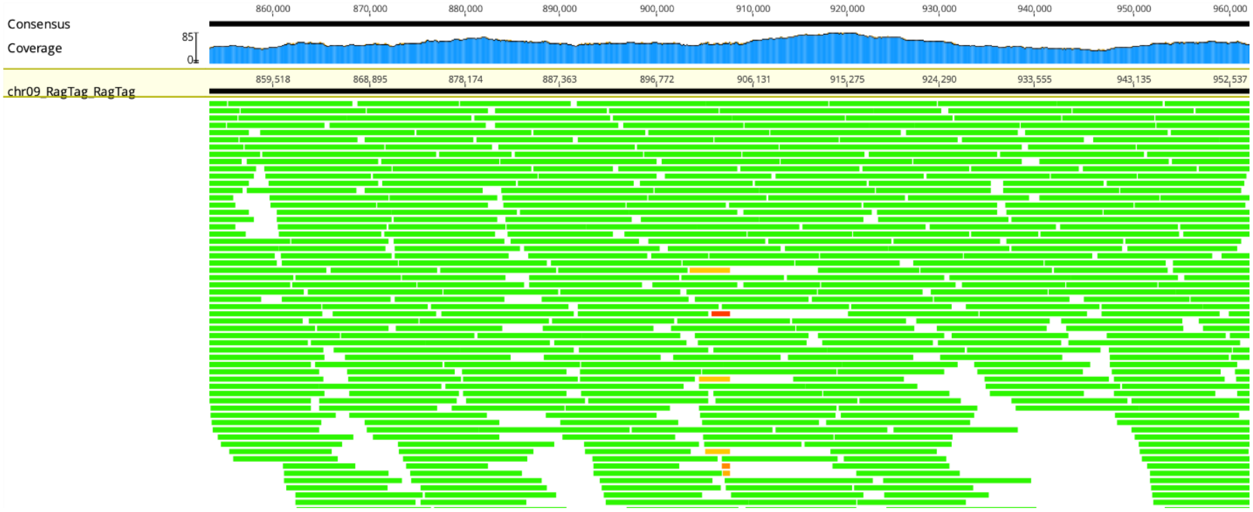

Supplementary Fig. 19. Long read coverage for MR1 *fom1* tandem duplication.
