# Supplementary material for: Graph-based pangenomics maximizes genotyping density and reveals structural impacts on fungal resistance in melon
Source: Nat Commun. 2022 Dec 22;13:7897. doi: 10.1038/s41467-022-35621-7 (PMC9780226; doi:10.1038/s41467-022-35621-7)
Supplement: Supplementary file 3 — Description of Additional Supplementary Files [file 41467_2022_35621_MOESM3_ESM.pdf]

Description of Additional Supplementary Files

Supplementary Data 1: Gaps in one genome relative to another for pairwise comparisons between all genomes in graph. 'N's are counted as gaps if aligned with an explicit base.

Supplementary Data 2: Primer specifications for *fom2* structural variation confirmation.
