# Supplementary material for: Graph-based pangenomics maximizes genotyping density and reveals structural impacts on fungal resistance in melon
Source: Nat Commun. 2022 Dec 22;13:7897. doi: 10.1038/s41467-022-35621-7 (PMC9780226; doi:10.1038/s41467-022-35621-7)
Supplement: Supplementary file 6 — Reporting Summary [file 41467_2022_35621_MOESM6_ESM.pdf]

Corresponding author(s): Justin Vaughn, Pat Wechter

Last updated by author(s): Jul 14, 2022

Reporting Summary

Nature Portfolio wishes to improve the reproducibility of the work that we publish. This form provides structure for consistency and transparency in reporting. For further information on Nature Portfolio policies, see our Editorial Policies and the Editorial Policy Checklist.

Statistics

For all statistical analyses, confirm that the following items are present in the figure legend, table legend, main text, or Methods section.

n/a	Confirmed	
☐	☒	The exact sample size (*n*) for each experimental group/condition, given as a discrete number and unit of measurement
☐	☒	A statement on whether measurements were taken from distinct samples or whether the same sample was measured repeatedly
☐	☒	The statistical test(s) used AND whether they are one- or two-sided<br>*Only common tests should be described solely by name; describe more complex techniques in the Methods section.*
☐	☒	A description of all covariates tested
☐	☒	A description of any assumptions or corrections, such as tests of normality and adjustment for multiple comparisons
☐	☒	A full description of the statistical parameters including central tendency (e.g. means) or other basic estimates (e.g. regression coefficient) AND variation (e.g. standard deviation) or associated estimates of uncertainty (e.g. confidence intervals)
☐	☒	For null hypothesis testing, the test statistic (e.g. *F*, *t*, *r*) with confidence intervals, effect sizes, degrees of freedom and *P* value noted<br>*Give P values as exact values whenever suitable.*
☒	☐	For Bayesian analysis, information on the choice of priors and Markov chain Monte Carlo settings
☒	☐	For hierarchical and complex designs, identification of the appropriate level for tests and full reporting of outcomes
☐	☒	Estimates of effect sizes (e.g. Cohen's *d*, Pearson's *r*), indicating how they were calculated

Our web collection on statistics for biologists contains articles on many of the points above.

Software and code

Policy information about availability of computer code

Data collection	None
Data analysis	All custom software is available via the open-source repository: https://github.com/USDA-ARS-GBRU/PanPipes.<br>Additional software: SMRTLink V8.0, BBTools (v38.87), BUSCO(v4), Jellyfish (v2.2.10), GenomeScope v2.0, Kmasker v1.1.1, bedtools v2.27.1, Braker v2.1.6 , TSEBRA v1.0.2, progressiveMauve (2015-02-13 linux release), vgtools (v1.37.0), bcl2fastq v2.20, bcftools v1.14, bwa v0.7.17-r1188, TASSEL v5.0, GAPIT3 (v2020.10.24), R (v3.4.1), Alphafold v2.0.0

For manuscripts utilizing custom algorithms or software that are central to the research but not yet described in published literature, software must be made available to editors and reviewers. We strongly encourage code deposition in a community repository (e.g. GitHub). See the Nature Portfolio guidelines for submitting code & software for further information.

Data

Policy information about availability of data

All manuscripts must include a data availability statement. This statement should provide the following information, where applicable:

- Accession codes, unique identifiers, or web links for publicly available datasets
- A description of any restrictions on data availability
- For clinical datasets or third party data, please ensure that the statement adheres to our policy

Raw data and genome assemblies have been deposited to NCBI under Bioproject accession PRJNA844271. Outside data sources used: NCBI Bioprojects PRJNA358655 and PRJNA358674.

Human research participants

Policy information about studies involving human research participants and Sex and Gender in Research.

Reporting on sex and gender	NA
Population characteristics	NA
Recruitment	NA
Ethics oversight	NA

Note that full information on the approval of the study protocol must also be provided in the manuscript.

Field-specific reporting

Please select the one below that is the best fit for your research. If you are not sure, read the appropriate sections before making your selection.

☒ Life sciences ☐ Behavioural & social sciences ☐ Ecological, evolutionary & environmental sciences

For a reference copy of the document with all sections, see nature.com/documents/nr-reporting-summary-flat.pdf

Life sciences study design

All studies must disclose on these points even when the disclosure is negative.

Sample size	Number of samples (n=149) was chosen in keeping with standard structured genetic experiments.
Data exclusions	Two individual lines were excluded from error rate analysis because of poor genotyping. They were included in additional aspects as described in manuscript.
Replication	Replication is not applicable to this study. Measurements were used for association but those have been defined in previous studies as described in manuscript. P-values generated in genome-wide association analyses are based on standard linear regression statistics (using lm() function in R) and represent the probability that a genomic variant does not co-vary with the trait of interest.
Randomization	NA, see above
Blinding	NA, no group effects were assessed

Reporting for specific materials, systems and methods

We require information from authors about some types of materials, experimental systems and methods used in many studies. Here, indicate whether each material, system or method listed is relevant to your study. If you are not sure if a list item applies to your research, read the appropriate section before selecting a response.

Materials & experimental systems

n/a	Involved in the study
☒	☐ Antibodies
☒	☐ Eukaryotic cell lines
☒	☐ Palaeontology and archaeology
☒	☐ Animals and other organisms
☒	☐ Clinical data
☒	☐ Dual use research of concern

Methods

n/a	Involved in the study
☒	☐ ChIP-seq
☒	☐ Flow cytometry
☒	☐ MRI-based neuroimaging
